# Designed Chemical Intervention with Thiols for Prophylactic Contraception

**DOI:** 10.1371/journal.pone.0067365

**Published:** 2013-06-27

**Authors:** Monika Sharma, Lokesh Kumar, Ashish Jain, Vikas Verma, Vikas Sharma, Bhavana Kushwaha, Nand Lal, Lalit Kumar, Tara Rawat, Anil K. Dwivedi, Jagdamba P. Maikhuri, Vishnu L. Sharma, Gopal Gupta

**Affiliations:** 1 Division of Endocrinology, CSIR-Central Drug Research Institute, Lucknow, India; 2 Division of Medicinal and Process Chemistry, CSIR-Central Drug Research Institute, Lucknow, India; 3 Division of Pharmaceutics, CSIR-Central Drug Research Institute, Lucknow, India; University of Hyderabad, India

## Abstract

Unlike somatic cells, sperm have several-fold more available-thiols that are susceptible to redox-active agents. The present study explains the mechanism behind the instant sperm-immobilizing and trichomonacidal activities of pyrrolidinium pyrrolidine-1-carbodithioate (PPC), a novel thiol agent rationally created for prophylactic contraception by minor chemical modifications of some known thiol drugs. PPC, and its three derivatives (with potential active-site blocked by alkylation), were synthesized and evaluated against live human sperm and metronidazole-susceptible and resistant *Trichomonas vaginalis*, *in vitro*. Sperm hexokinase activity was evaluated by coupled enzyme assay. PPC irreversibly immobilized 100% human sperm in ∼30 seconds and totally eliminated *Trichomonas vaginalis* more efficiently than nonoxynol-9 and metronidazole. It significantly inhibited (P<0.001) thiol-sensitive sperm hexokinase. However, the molecule completely lost all its biological activities once its thiol group was blocked by alkylation. PPC was subsequently formulated into a mucoadhesive vaginal film using GRaS excipients and evaluated for spermicidal and microbicidal activities (*in vitro*), and contraceptive efficacy in rabbits. PPC remained fully active in quick-dissolving, mucoadhesive vaginal-film formulation, and these PPC-films significantly reduced pregnancy and fertility rates in rabbits. The films released ∼90% of PPC in simulated vaginal fluid (pH 4.2) at 37°C in 5 minutes, *in vitro*. We have thus discovered a common target (reactive thiols) on chiefly-anaerobic, redox-sensitive cells like sperm and *Trichomonas*, which is susceptible to designed chemical interference for prophylactic contraception. The active thiol in PPC inactivates sperm and *Trichomonas* via interference with crucial sulfhydryl-disulfide based reactions, e.g. hexokinase activation in human sperm. In comparison to non-specific surfactant action of OTC spermicide nonoxynol-9, the action of thiol-active PPC is apparently much more specific, potent and safe. PPC presents a proof-of-concept for prophylactic contraception via manipulation of thiols in vagina for selective targeting of sperm and *Trichomonas*, and qualifies as a promising lead for the development of dually protective vaginal-contraceptive.

## Introduction

The transmission of fertile spermatozoa and sexually transmitted disease (STD) pathogens during heterosexual contacts may lead to frequent unwanted pregnancies (mostly ending in abortions) [1], and infections. Trichomoniasis, the most prevalent non-viral STD, predisposes women to viral STDs, HIV/AIDS and cervical cancer; and newborns to pre-term delivery, low birth weight and high mortality rate [2]. Prophylactic contraceptives targeting both sperm and *Trichomonas* could be an ideal strategy to prevent the heterosexual spread of trichomoniasis since contraception is desired during majority of sexual acts. Unfortunately metronidazole, (the FDA-approved drug against *Trichomonas vaginalis*) lacks contraceptive activity, has insufficient intra-vaginal efficacy [3] and proves ineffective against resistant *Trichomonas* [4]. Some non-imidazoles have shown intra-vaginal potency against metronidazole-resistant *Trichomonas* infection [5], but are devoid of contraceptive activity. Nonoxynol-9 (a non-ionic detergent), which forms the active ingredient in most OTC spermicides, kills sperm and STD pathogens (including *Trichomonas*) by its non-specific, surfactant action. However, clinical trials have shown that repeated use of N-9 containing vaginal products could harm the vaginal mucosa and increase susceptibility to STDs, including HIV [6], [7].

According to an estimate, human sperm contain >55 nmoles of ‘reactive’ thiols per 10^8^ cells which are ∼30 times more than those on erythrocytes [8]. The significance of thiols in sperm cell motility/function is evident from the fact that asthenozoospermic infertile men have significantly less thiols on sperm than normozoospermic men [9]. It has already been well established that the motility and metabolism of sperm can be inhibited substantially by agents having affinity for sulfhydryls, the effect being reversible only negligibly in some cases by cysteine and glutathione [10]. Equally important, *T. vaginalis* lacks glutathione (the intracellular redox buffer), glutathione dependent peroxidase, and catalase, and therefore it relies heavily on cysteine (which constitutes >70% of cell’s total thiol pool) for protection against redox-stress, making it extremely susceptible to sulfhydryl-manipulating agents [11]. Thus, exploiting thiols as a common target on both sperm and *Trichomonas* we designed several dually active, non-surfactant molecular prototypes for prophylactic contraception [12]–[17]. However, a perfect balance of the two activities could not be achieved optimally. Nevertheless, our recent efforts in this direction has yielded a valuable series of dually-active molecules and the most promising structure (pyrrolidinium pyrrolidine-1-carbodithioate, PPC) instantly inactivated 100% human sperm more efficiently and specifically than N-9, and completely eliminated *Trichomonas vaginalis* (resistant and susceptible strains) more potently than metronidazole, *in vitro* [18]. The human sperm permanently paralyzed by PPC had considerably reduced numbers of free thiols [18]. We now present data to pinpoint the active site on the molecule and its potential interference with the activity of a thiol-sensitive, rate-limiting key enzyme of the sperm energy metabolism. Yet, the microbicidal and contraceptive relevance of such molecules depend on their vaginal activity and stability in appropriate formulations designed for their pre-coital intravaginal delivery to prevent conception and disease. Consequently we have also formulated the compound into a convenient contraceptive product (vaginal film) and have studied its efficacy using suitable *in vitro* (human) and *in vivo* (animal) models.

## Materials and Methods

### Materials

**Figure 1:**
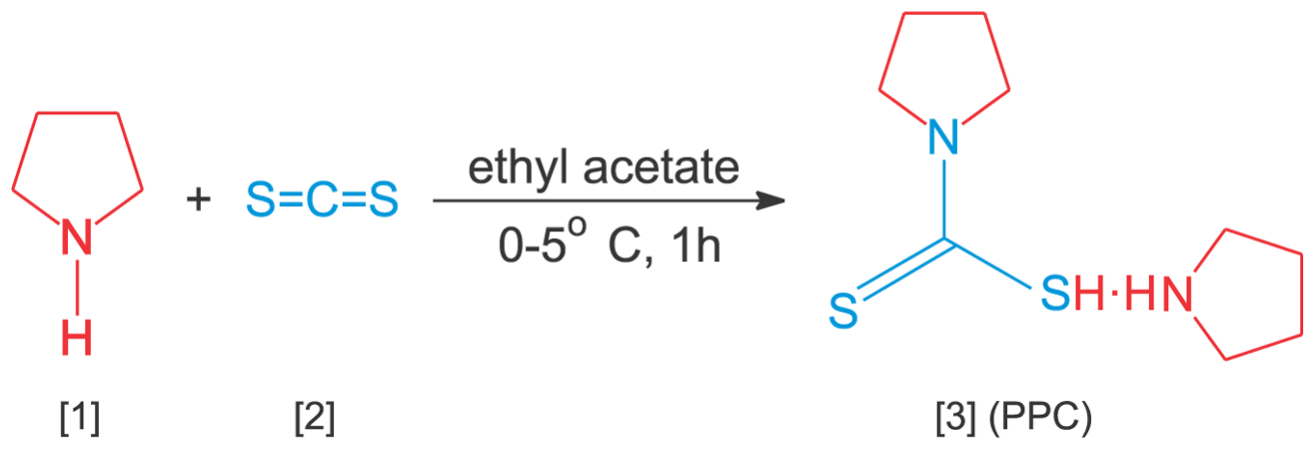
The synthesis of pyrrolidinium pyrrolidine-1-carbodithioate (PPC, 3) from pyrrolidine (1) and carbon disulfide (2).

P***yrrolidinium pyrrolidine-1-carbodithioate***, (PPC) and its derivatives (with active site protected by alkylation) were synthesized and characterized by the authors NL, LK, TR and VLS. Pyrrolidine, carbon disulfide, metronidazole, hydroxypropylmethylcellulose (HPMC), hydroxyethyl cellulose (HEC), polyvinylalcohol (PVA), chitosan (CH) and acetonitrile were from Sigma-Aldrich, St. Louis, MO, USA; sodium salt of octane sulfonic acid, phosphoric acid, polyethylene glycol (PEG 400) were procured from Merck, Germany; and nonoxynol-9 was from Spectrum Chemical Manufacturing Corp. (New Brunswick, NJ, USA). All other bio-chemicals, substrates, enzymes were from Sigma-Aldrich, unless stated otherwise. All solutions were prepared in Milli-Q (18.2 MΩ.cm) water. PPC was synthesized by the reaction of pyrrolidine (**1**) with carbon disulfide (**2**) at 0–5°C in ethylacetate (Figure 1). Details of synthesis and physicochemical characterization are provided as supporting data (Figure S1 in File S1). Derivatives of PPC were prepared by alkylation of its active thiol. Three structures, benzyl pyrrolidine-1-carbodithioate (BPC); isobutyl pyrrolidine-1-carbodithioate (IPC) and 2-hydroxyethyl pyrrolidine-1-carbodithioate (HPC) were synthesized by reacting PPC with appropriate alkyl halide and triethyl amine in methanol at room temperature for 3–4 hours to furnish compounds in good yield. The detailed chemistry and characterization of compounds is furnished as supporting information (Figure S3 in File S1).

### Human Sperm and Spermicidal Assay

Freshly ejaculated human semen was obtained by masturbation from healthy and normal volunteers and allowed to liquefy at 37°C for 45 min. Semen characteristics and analysis were performed according to the normal criteria as per World Health Organization guidelines [19]. Sperm count and motility analysis were performed manually as well as in a Computer Automated Semen Analyzer (CASA) system using a small drop of liquefied semen placed on a ‘Makler’ counting chamber (Sefý Medica, Hafia, Israel) pre-warmed to 37°C. Semen samples with >65 million/ml sperm count, >70% motility and normal sperm morphology were used. This study was approved by the Institutional Ethics Committee for Human Research and prior informed written consent was obtained from the donors for this study. The spermicidal assay of pure compound has been reported earlier [17]. The minimum effective (spermicidal) concentration (MEC) of the active ingredient (PPC) in vaginal film was determined by the modified Sander-Cramer assay [14], [20]. Briefly, the test films were dissolved in SVF [21] to make a 50.0 mM solution of the active ingredient and then serially diluted to 0.125 mM with saline. A spermicidal test was performed with each solution starting from 50 mM until the MEC was achieved. For this purpose, 0.05 ml of liquefied human semen was added to 0.25 ml of test solution and mixed gently for 10 s at a low speed. A drop of the mixture was then immediately examined under a phase-contrast microscope for 100% immobilization of spermatozoa in the next ∼20 s, and the immobility continuing even after dilution with 1.0 ml of Krebs Ringer bicarbonate buffer for another 30 min at 37°C. The MEC was determined in at least three individual semen samples from different donors. The minimum concentration of compound capable of immobilizing 100% sperm in ∼30 s in all the semen samples was denoted as the MEC.

### *T. vaginalis* Cultures and Trichomonacidal Assay

Clinical isolates of metronidazole-susceptible *T. vaginalis* collected at Post Graduate Institute of Medical Research and Education, Chandigarh, India, were obtained from the laboratory of Divya Singh (CSIR-CDRI, Lucknow, India), and a metronidazole-resistant strain of *T. vaginalis* (CDC085 [ATCC 50143]) was procured from the American Type Culture Collection (ATCC). Both strains were cultured under partial anaerobic condition in TYM medium as detailed earlier [18]. Organisms in the logarithmic phase of growth and exhibiting motility and normal morphology were harvested, centrifuged, and resuspended in fresh TYM medium for the experiments. *In vitro* drug susceptibility assays were carried out according to the standard procedure [22] and the metronidazole susceptibility criteria of Sobel et al. [23] was used to determine the resistance of *T. vaginalis* strains to metronidazole. Accordingly, the clinical isolate was categorized as susceptible, and the ATCC strain was categorized firmly resistant. The vaginal films were dissolved in SVF to make a 10.0 mM solution of PPC (active ingredient) and diluted with TYM medium serially to 1.0 µM in a 48-well plate. Placebo films were processed similarly and used as vehicle in the control wells. Parasites (5 X 10^3^ trophozoites/well) were added to these wells and incubated anaerobically at 37°C. Trophozoite growth and viability in drug-containing wells were monitored by trypan blue staining and cell number score on a daily basis, in comparison to the control. Assay results were clearly defined after 48 h in terms of the MIC (the lowest concentration of compound at which all trophozoites were nonviable). Viability was determined by trypan blue exclusion and 100% eradication was confirmed by transferring 100 µl of the suspension to a 15-ml tube with fresh medium and recording the growth at 37°C for 14 days [24] **.** Metronidazole (1 to 39 µM susceptible strain; 40 to 400 µM resistant strain) was used as reference standard. Three separate experiments were performed for each strain to confirm the MIC.

### Sperm Hexokinase Assay

Hexokinase activity was assayed by enzyme coupled reaction method 25. Normal, motile, human sperm were pelleted from semen samples (that were used in spermicidal assays), washed with medium-199 and treated (in medium-199) with PPC, BPC, IPC or HPC separately at 150 µM concentration for 2 minutes. Sperm samples taken parallel in control tubes were treated with 0.05% DMSO. After incubation, sperm were pelleted at 4.0°C, washed 2 to 3 times with ice-cold PBS and resuspended in RIPA buffer (containing Tris 50 mM, NaCl 150 mM, Triton X-100 1.0%, SDS 2%, sodium deoxycholate 0.5%, Sodium orthovanadate 1 mM, PMSF 1 mM, sodium fluoride 5 mM, pH 8.0), incubated for 2 hour at 4°C and homogenized by sonication for 2 min at 4°C. The assay solution (150 µl) contained 20 mM Tris-HCl, 20 mM MgCl_2_, 4 mM EDTA, one unit/ml glucose 6-phosphate dehydrogenase, 10 mM glucose, 0.6 mM β-NADP^+^, and 0.1% Triton X-100 (pH 7.6). 5.0 µL of sperm homogenate containing ∼10–20 µg protein was added to the assay mixture and after preincubation of 3 min at 30°C, enzyme reaction was initiated by adding 4.0 mM ATP. Formation of NADPH^+^ was monitored at 340 nm and ΔOD was calculated during the linear reaction time. Sample protein was determined by Bradford method.

### Preparation of Mucoadhesive Vaginal Films Containing PPC and Evaluation of its Physical Properties

**Figure 2:**
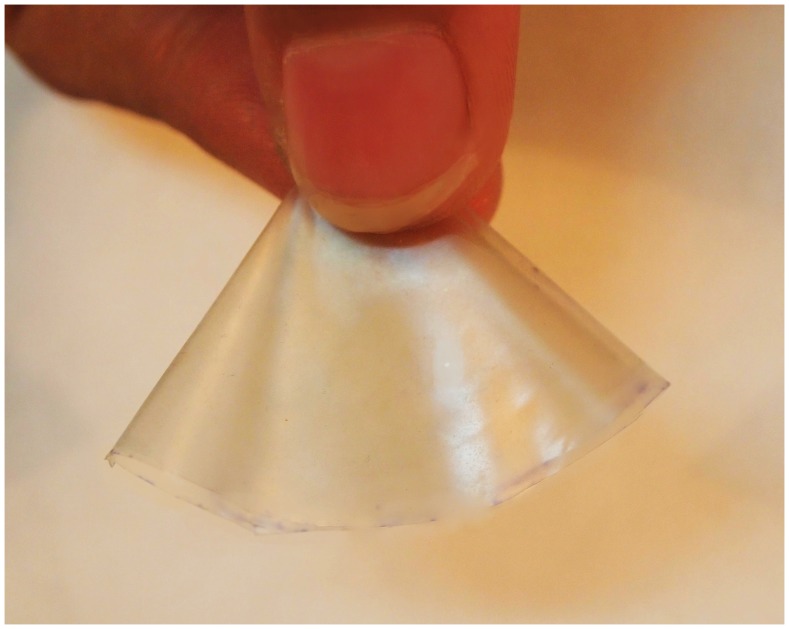
Mucoadhesive vaginal contraceptive film incorporating pyrrolidinium pyrrolidinium pyrrolidine-1-carbodithioate (PPC) as active ingredient.

Mucoadhesive films were prepared by film casting method using mucoadhesive polymers namely hydroxypropyl methylcellulose (HPMC), hydroxyethyl cellulose (HEC), polyvinyl alcohol (PVA) and chitosan (CH) in different combinations. Two percent solution of each polymer (HPMC, HEC and PVA) was made in water while 2% chitosan solution also contained 1% v/v acetic acid. Polyethylene glycol (PEG 400) was used as solubilizer and plasticizer in the film casting solution. Mixture of a definite ratio of each polymer solution was vortexed, and 15, 20 or 25 mg compound (PPC) dissolved in propylene glycol was incorporated into the polymeric solution. The medicated casting solutions were then left overnight at room temperature to ensure clear, bubble-free gel. The gel was cast into glass petri dish and allowed to dry at 40°C until a flexible film was formed (Figure 2). The dried films were packed in aluminium foil, stored in a dessicator at room temperature and used for *in vitro* and *in vivo* evaluations. **Physical properties:** ***Weight*** **:** Average weight of six films was determined with standard deviation; ***Thickness*** **:** The thickness of each film was measured at five different locations (center and four places at circumference) using a micrometer screw gauge to obtain the mean value; ***Swelling index*** **:** The films were coated on the lower side with ethyl cellulose (to avoid sticking to the dish) then weighed (W1) and placed separately in petri dishes containing 10 ml of distilled water at room temperature. After 20 minutes, films were removed and dried using filter paper. The swollen discs were weighed (W2) and the percentage of swelling was calculated by the following formula, Swelling index = (W2-W1/W1) * 100.

### In vitro Release of PPC from Vaginal Films

The release of compound (PPC) from the bioadhesive films into SVF (pH 4.2) at 37±0.5°C was estimated using modified Levy method. Briefly, each bioadhesive film was adhered to the side wall of a vessel using cyanoacrylate and SVF was placed in the vessel. The vessel was covered and fitted with a magnetic stirrer. At each time interval a sample was withdrawn, filtered through a millipore filter (0.45 µm) and analyzed by HPLC. A similar volume of SVF was added to the release medium to maintain the volume in the vessel constant.

### Contraceptive Efficacy of PPC Vaginal Films in Rabbits

Adult female Belgian rabbits were given intravaginal instillation of vaginal films containing 0 (placebo), 15, 20 and 25 mg PPC. For this, the films were dissolved in 3.0 ml saline and instilled intravaginally with the help of a 15 cm long catheter attached to a syringe. After 10 min, the treated females were mated once with a fertile buck and kept in separate cages. A total of 10 proven fertile male rabbits were randomly used in this experiment and a male was mated only once on a given occasion. The mating was confirmed by the presence of sperm in the vaginal smear and the mated females were allowed to complete gestation (30–35 days). At delivery, the number of pups born was recorded.

### Statistical Analysis

Where necessary, the results were analyzed by one-way analysis of variance (ANOVA) and P values less than 0.05 were considered as significant.

### Ethical Approvals

The use of semen samples from young, adult, normal human volunteers for *in vitro* spermicidal assays and mechanism of action studies was approved by the Institutional Ethics Committee for Human Research of the CSIR-Central Drug Research Institute, Lucknow, India, and informed written consent was obtained from the donors. The *in vivo* study of contraceptive efficacy in rabbits was approved by the Institutional Animal Ethics Committee vide approval No. IAEC/2008/56/Renewed 30.11.2011.

## Results

### Effect of Sulfhydryl-blocking by Alkylation of PPC Molecule

**Figure 3:**
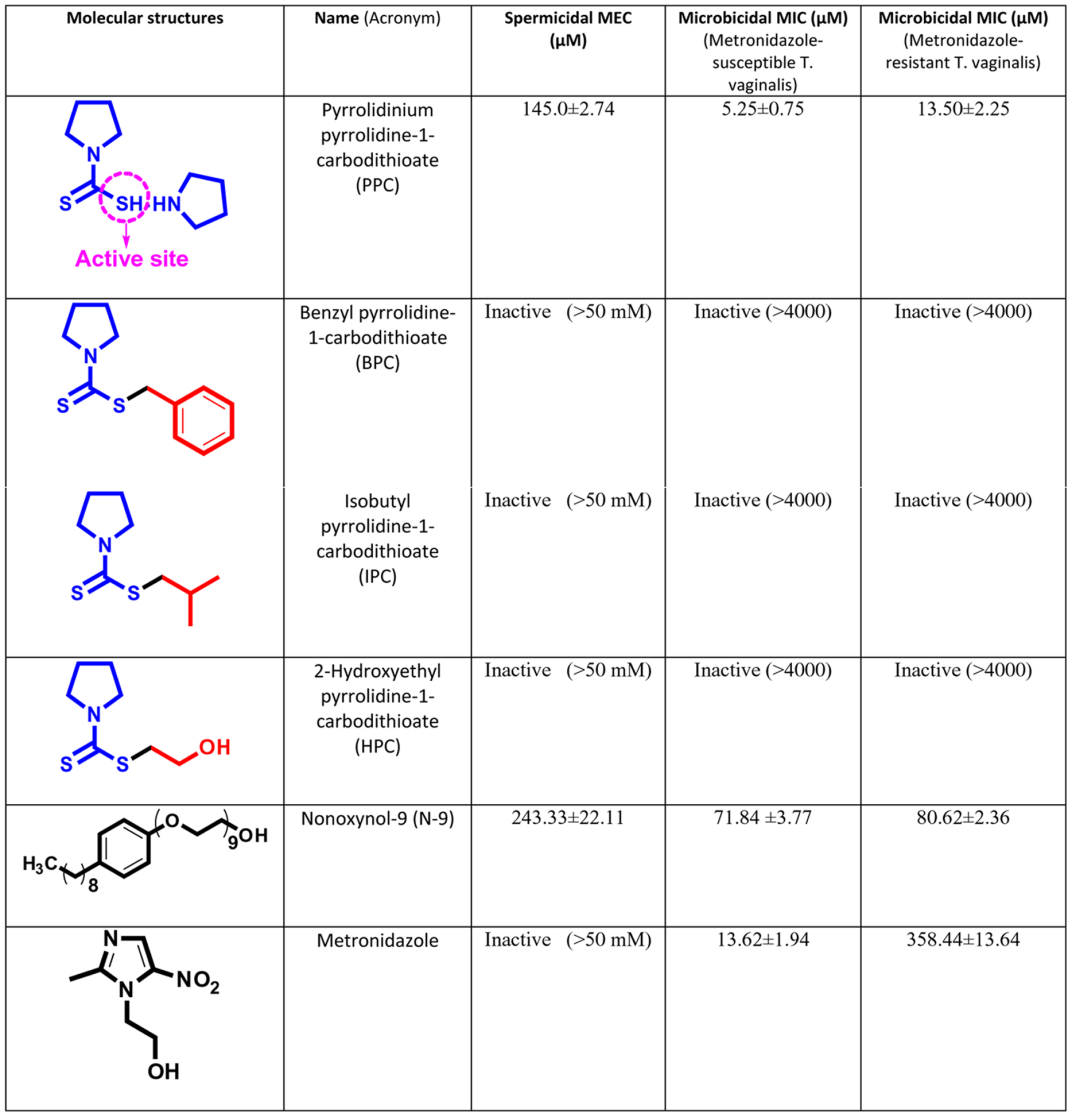
The *in vitro* spermicidal and anti-trichomonal activities of pyrrolidinium pyrrolidine-1-carbodithioate (PPC) and its derivatives (BPC, IPC & HPC) with the active thiol blocked by alkylation. (Mean ± SE of three independent experiments; MEC = minimum effective concentration; MIC = minimum inhibitory concentration).

PPC exerted a permanent paralyzing effect on human sperm at ∼150 µM as reported by us earlier [18], an effect plausibly caused by the interaction of its proactive thiol with available thiols on sperm. To conclusively prove this point, we blocked the PPC’s active thiol by alkylation, making it unavailable for reaction. PPC’s derivatives with active site blocked (benzyl pyrrolidine-1-carbodithioate [BPC]; isobutyl pyrrolidine-1-carbodithioate [IPC]; 2-hydroxyethyl pyrrolidine-1-carbodithioate [HPC]) completely failed to exhibit any sperm immobilizing and anti-trichomonal activities (Figure 3).

### Effect of PPC and its Derivatives on Human Sperm Hexokinase – a Thiol Sensitive Enzyme

**Figure 4:**
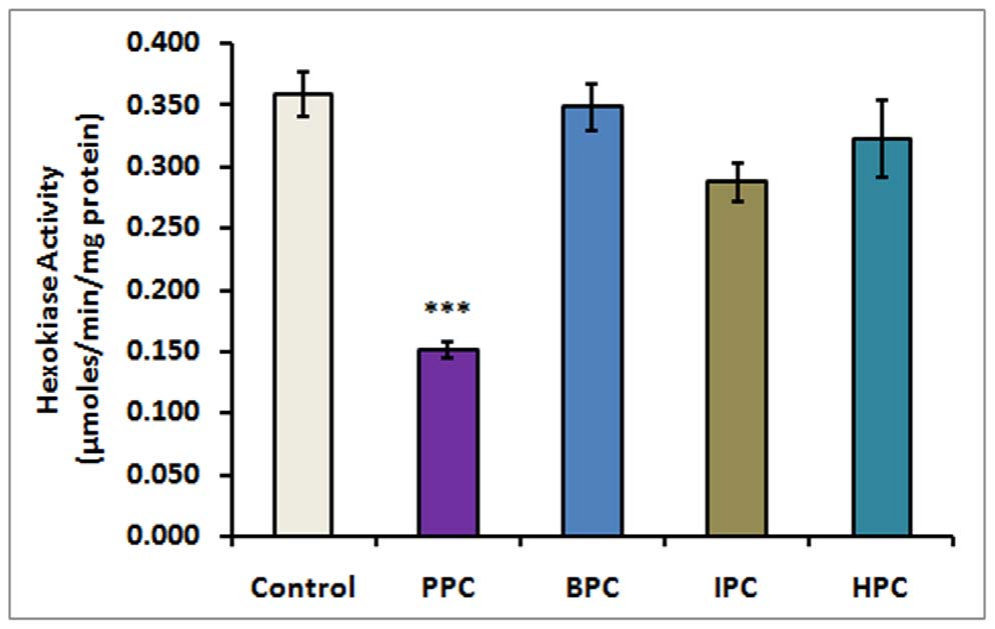
Hexokinase activity of human sperm treated with 0 (control) and 150 µM of PPC and its derivatives BPC, IPC and HPC. (Mean ± SE of three independent experiments, ***P<0.001).

Hexokinase in human sperm exhibited an average specific activity of 359±18 nmoles.min^−1^.mg-protein^−1^ that was inhibited significantly by ∼58% to 152 nmoles.min^−1^.mg-protein^−1^ (P<0.001) in sperm permanently paralyzed with spermicidal concentration of PPC. Human sperm samples treated parallel with same concentration of PPC derivatives did not exhibit any significant reduction in hexokinase activity (Figure 4). Sperm samples treated with BPC, IPC and HPC exhibited specific activities of 348±19, 287±15 and 322±31 nmoles.min^−1^.mg-protein^−1^, respectively, which were not significantly different from the control value.

### Optimized Film-formulation for Vaginal Delivery of PPC

**Figure 5:**
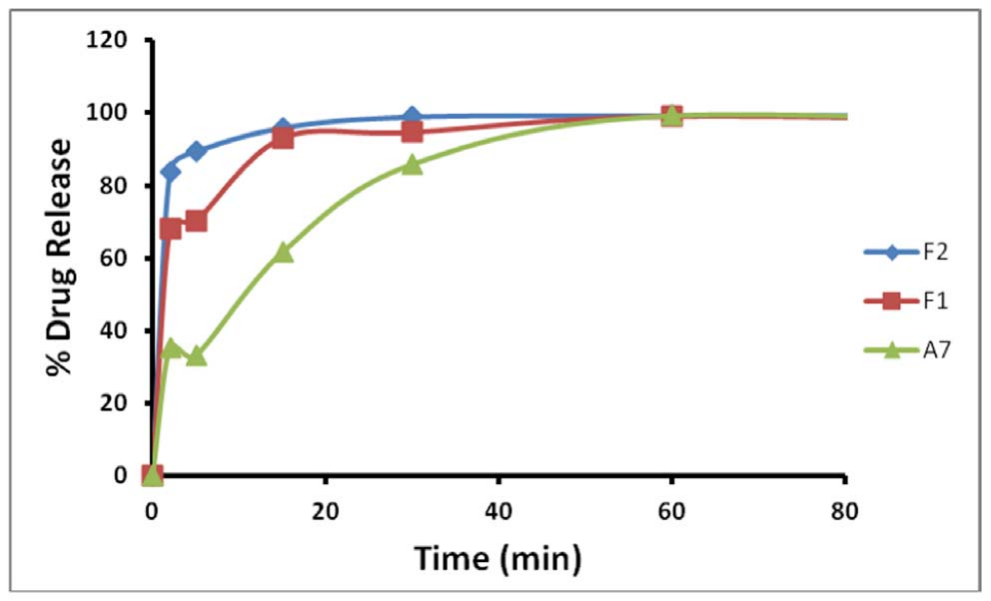
Pyrrolidinium pyrrolidine-1-carbodithioate (PPC) release from mucoadhesive vaginal contraceptive film-formulations F1, F2 and A7, in simulated vaginal fluid (SVF) at 37°C.

Several combinations of GRAS (Generally Recognized as Safe) polymers were experimented with to obtain the optimum vaginal film formulation for the new spermicidal compound (Table S2 in file S1). Three formulations A7, F1 and F2 were finally shortlisted on the basis of desired physical properties (Table 1). A contraceptive film is intended to be folded twice before vaginal insertion; consequently the soft and flexible nature of formulations F1 and F2 was preferred over the somewhat papery texture of A7 that gave sharp corners on folding. Furthermore, F2 released 83% of PPC in SVF at 37°C during initial 2 minutes and more than 95% of compound was released by 15 min, while the compound released in 15 min was 93% and 61% from formulations F1 and A7, respectively (Figure 5). Formulations F1 and F2 gave thinner, quicker-dissolving films than formulation A7. Hence formulation F2 was selected for all *in vitro* and *in vivo* efficacy studies.

**Table 1:** Composition and physical characteristics (Mean±SD) of vaginal film formulations incorporating PPC.

Film formulation	Polymer ratio					PPC(mg)	Thickness(mm)	Weight(g)	Swelling index	Physical properties
	CH(2%)	HEC(2%)	PVA(2%)	HPMC(2%)	PEG(%)					
**A7**	1	2	1	0	5.64	20	0.77±0.007	1.27±0.05	54%	Firm, papery, translucent
**F1**	0	1	0.01	1	5.0	20	0.42±0.001	0.87±0.10	eroded	Soft, flexible, transparent
**F2**	**0**	**1**	**0**	**1**	**2.25**	**20**	**0.44±0.001**	**0.70±0.06**	**eroded**	**Soft, flexible, transparent**

PEG-400 was used as a solubilizer and plasticizer at a final concentration of 2.25 to 5.64%.

### In vitro Efficacy of PPC in Vaginal Film Formulation

The spermicidal MEC of PPC in vaginal film formulation was **149.0±3.67**µM**.** In the same formulation, PPC exhibited anti-Trichomonal MICs of **9.37±1.56 **µM and **83.33±20.8 **µM respectively. On the other hand, it retained good compatibility with vaginal microflora (*Lactobacillus acidophilus*) and human cervical (HeLa) cells with a high IC_50_ of **743.1±18.3** and **1096±28 **µM respectively, in viability assays. The placebo film exerted no effect on the viabilities of sperm, *Trichomonas vaginalis HeLa* cells and *L. acidophilus*, *in vitro* (Table 2).

**Table 2:** The spermicidal and microbicidal activities of pyrrolidinium pyrrolidine-1-carbodithioate (PPC) in vaginal film formulation and its compatibility with human cervical HeLa cells and *Lactobacillus acidophilus*, *in vitro*.

Test material	PPC content (mg)	Spermicidal MEC of active ingredient (PPC)	Microbicidal MIC of active ingredient (PPC) against *T. vaginalis* (met-susceptible)	Microbicidal MIC of active ingredient (PPC) against *T. vaginalis* (met-resistant)	Compatibility with HeLa (IC_50_)	Compatibility with *Lactobacillus acidophilus* (IC_50_)
**PPC film**	20.0	149.0±3.67 µM	9.37±1.56 µM	83.33±20.8 µM	1096±28 µM	743.1±18.3 µM
**Placebo film**	0	No effect	No effect	No effect	No effect	No effect

(Mean ± SE of 3 independent experiments; MEC = minimum effective concentration; MIC = minimum inhibitory concentration).

*In vitro* microbicidal MIC of pure metronidazole = ∼11 & 340 µM against susceptible and resistant strains of *T. vaginalis*, respectively.

### Contraceptive Efficacy of PPC Based Vaginal Films in Rabbits

Female rabbits which received vaginal instillation of 0 (placebo), 15, 20 and 25 mg of PPC in film formulation before mating exhibited 0, 60, 60 and 75% reduction in pregnancy rates with 5/5, 2/5, 2/5 and 1/4 rabbits becoming pregnant in these groups, respectively. Concomitantly, the fertility rates were inhibited by 66, 74 and 91% in these rabbits as the average litter size got reduced from 5.4 in placebo (0 mg) group to 1.8, 1.4 and 0.5 in 15, 20 and 25 mg PPC groups, respectively (Table 3).

**Table 3:** *In vivo* contraceptive efficacy of PPC vaginal films in rabbits.

Treatment	Rabbits pregnant	Contraceptive Efficacy (%)	Average litter size	Fertility Inhibition (%)
Placebofilm	5/5	0	5.4	0
15 mg PPC film	2/5	60	1.8	66
20 mg PPC film	2/5	60	1.4	74
25 mg PPC film	1/4	75	0.5	91

## Discussion

Pyrrolidinium pyrrolidine-1-carbodithioate (PPC), a novel compound designed to inhibit free thiols, behaved as a potent Trichomonacidal spermicide which significantly inhibited the thiol sensitive hexokinase in human sperm. On the other hand, PPC lost all its biological activities when its active site was blocked by alkylation. However, the compound retained its contraceptive potential in vaginal film formulation and significantly inhibited pregnancy in rabbits.

PPC was evolved from dithiocarbamates that have been used in health care for the management of alcoholism and heavy metal poisoning [26], HIV infection [27], cancer [28], and atherosclerosis [29]. Most of these compounds have well established safety for human use through oral medication. However, their topical sperm immobilizing action was found to be moderate and weaker than the OTC spermicide nonoxynol-9 (data not included). However, the sperm immobilizing capability of these dithiocarbamates could be potentiated significantly by improving the redox potential of their sulfhydryls. Consequently, we replaced the strong electropositive sodium ion in pyrrolidine dithiocarbamate sodium salt [30] with a weaker base (amine), creating a novel thiol-agent PPC, in which the charge-transfer interactions between pyrrolidine and sulfhydryl groups kept the sulfhydryl intact and made it readily available for interaction with sperm thiols. This modification potentiated the spermicidal activity of PPC by ∼100 fold over the parent structure and made it even more active than N-9 [18]. To further prove this supposition, in the present study we designed derivates of PPC in which the sulfhydryl group was blocked by alkylation. All these molecules completely failed to show any sperm immobilizing and anti-trichomonal ability *in vitro*, which firmly established our hypothesis.

Sperm gain vigorous motility (which is crucial for fertility) as soon as they are released from the epididymis during ejaculation. This sudden burst of physical activity demands large amounts of energy that is provided chiefly by anaerobic glycolysis [31], [32]. The sperm specific hexokinase, which is a rate limiting glycolytic enzyme, is a thiol-sensitive protein that is activated by thiol-disulfide inter-conversions after ejaculation [25]. The presence of a thiol-sensitive active site on hexokinase has also been demonstrated in diaphragm, brain, testis and kidney of rat that is susceptible to disulfide poisoning [33]. We have already shown in our previous study that PPC treatment significantly alters the number of free thiols in human sperm [18]. Accordingly, we hypothesized that hexokinase could be one of the potential targets for this SH-binding, sperm immobilizing agent. PPC at its minimum spermicidal concentration inhibited human sperm hexokinase by >50%, and this could be one of the key mechanisms adding to its sperm immobilizing action. It is pertinent to note here that PPC derivatives (with active SH-group protected by alkylation) did not inhibit hexokinase activity and motility of human sperm. This may substantiate partially the spermicidal mechanism of PPC involving hexokinase, plausibly through alteration of sperm thiols. Similarly, it has been reported that inhibition of glycolytic enzymes by thiol disruption in *T. vaginalis* results in severe depletion of intracellular ATP [34] and that thiol-based redox systems play a decisive role in its survival [35]. In view of the emerging drug resistance crucial redox enzymes in these parasites have been projected as potential targets for new drug development [36]. PPC’s inherent design predisposes it to interfere with the thiol-based redox systems of susceptible micro-organisms like *Trichomonas vaginalis*, providing the required override on drug resistance [18]. In the present study PPC derivatives lacking thiol binding capacity failed as anti-trichomonal agents, which established thiols as a common target on sperm and *Trichomonas* for PPC.

Compounds capable of immobilizing human sperm rapidly and irreversibly at apparently safe concentrations *in vitro* may have contraceptive application. However, the utility of such molecules as topical contraceptives requires their stability and *in vivo* efficacy in formulations employed for their vaginal delivery. We have already published the *in vitro* efficacy and safety of PPC as a pure compound [18], and we have now attempted to establish its efficacy *in vivo,* in a suitable vaginal delivery system. Mucoadhesive vaginal contraceptive films are non-greasy/messy and more user-friendly delivery option than the conventional vaginal products such as gels and creams, which also require an applicator. A suitable film-formulation containing PPC was developed using GRAS excipients that solubilized rapidly in simulated vaginal fluid at 37°C and had excellent drug release profile as well as aesthetic appeal. The compound retained full activity as spermicide (against human sperm) in the film formulation, *in vitro*. In rabbit assay, the contraceptive films prevented pregnancy in 75% animals at 25 mg vaginal dose *in vivo*, reducing the overall fertility rate by 91%. Possibly, testing *in vivo* contraceptive activity in 8–9 females per dose (divided in 2–3 groups) could have furnished more precise assessment of the contraceptive efficacy of PPC. However, as per the guidelines of the Institutional Ethics Committee for Animal Experimentations we have used 4–5 animals per dose, which was a limitation of this study. Given that the fertility of rabbits far exceeds that of humans by having ∼100% conception rates (∼30% in humans), extremely high inseminating doses of sperm and multiple ovulations after mating [37]; even strong detergent based vaginal products fail to show 100% contraceptive efficacy in rabbit assays. Accordingly, a modest 30% reduction in fertility rate of rabbits has been reported with 25 mg N-9 in OTC vaginal cream formulation [38]. It is also pertinent to note that rabbit sperm have fewer thiols than human sperm [8], which could further weaken the sperm-paralyzing effects of –SH binding agents like PPC in rabbits. Yet, PPC exhibited an apparently higher contraceptive potential than the surfactant nonoxynol-9 under experimental conditions. On the other hand, PPC *per se* appeared more active against *Trichomonas vaginalis* [18] than in film formulation, which could be attributed partially to the observed changes in resistance of pathogens and interference of film constituents in viability assays. Nevertheless, PPC’s complementary trichomonacidal activity, stability at room temperature in vaginal delivery formulations and significant *in vivo* contraceptive efficacy makes it an ideal candidate for further development as a dually protective vaginal contraceptive. However, this would require its pharmacokinetic evaluation by vaginal route of administration followed by the mandatory local and systemic toxicity studies in suitable animal models, which have been planned. Though it may be argued that PPC should be active against several pathogenic microorganisms (bacteria, protozoa etc), to qualify as a microbicide. However, broad spectrum non-specific agents have proved to be ineffective due to the associated toxicity (6,7). On the other hand, specifically-acting anti-HIV microbicide Tenofovir, a reverse transcriptase inhibitor devoid of anti-bacterial and anti-protozoal activities, has renewed hope in the prophylactic approach of microbicides (39). Hence PPC may prove to be an effective microbicide by its specific action against *Trichomonas vaginalis,* which is known to increase susceptibility to other bacterial and viral STDs, including HIV (2).

## Supporting Information

File S1 The supporting Information contains physicochemical characterization data and synthesis details of PPC (Figure S1) and its derivatives (Figure S3); proposed reaction of PPC (Figure S2) and its derivatives (Figure S4) with sperm proteins; the spermicidal activity of reactants and the product PPC (Table S1); NMR spectra of PPC derivatives viz. BPC (Figure S5), IPC (Figure S6) and HPC (Figure S7); optimization details of film formulation for PPC (Table S2) and a typical HPLC chromatogram of PPC (Figure S8).
(PDF)
