# Supplementary material for: Designed Chemical Intervention with Thiols for Prophylactic Contraception
Source: PLoS One. 2013 Jun 27;8(6):e67365. doi: 10.1371/journal.pone.0067365 (PMC3694959; doi:10.1371/journal.pone.0067365)
Supplement: File S1 — The supporting Information contains physicochemical characterization data and synthesis details of PPC (Figure S1) and its derivatives (Figure S3); proposed reaction of PPC (Figure S2) and its derivatives (Figure S4) with sperm proteins; the spermicidal activity of reactants and the product PPC (Table S1); NMR spectra of PPC derivatives viz. BPC (Figure S5), IPC (Figure S6) and HPC (Figure S7); optimization details of film formulation for PPC (Table S2) and a typical HPLC chromatogram of PPC (Figure S8). (PDF) [file pone.0067365.s001.pdf]

Supporting Data

Synthesis of pyrrolidinium pyrrolidine-1-carbodithioate (PPC) and its derivatives

Instrumentation: IR spectra (ν_{max} in cm^{-1}) of the compounds were recorded on PerkinElmer Spectrum Version 10.03.06. ^1H NMR spectra were recorded on Bruker Supercon Magnet Avance DPX-200/DRX-300 spectrometers (operating at 200 and 300 MHz respectively for ^1H) in deuterated solvents with TMS as internal reference (chemical shifts δ in ppm, J in Hz.). Elemental analyses were performed on Carlo Erba EA-1108 micro analyzer / Vario EL-III C H N S analyzer. All compounds were analyzed of C, H, N and the results obtained were within ± 0.4% of calculated values. The reaction progress was routinely monitored by thin layer chromatography (TLC) on pre-coated alumina / silica gel plates (Aldrich).

Synthesis: The compound (**PPC**) was synthesized according to figure S1 where pyrrolidine (**1**) was reacted with carbon disulfide (**2**) at 0-5 °C in ethylacetate. Briefly, to a solution of pyrrolidine (16.5 mmol) in ethyl acetate (20 mL) carbon disulfide (8.25 mmol) was added drop-wise within 30 minutes with stirring at 0-5°C. The reaction mixture was further stirred at 0-5°C for 30 min. A white solid that separated was filtered off and washed with ethyl acetate to give the compound-PPC.

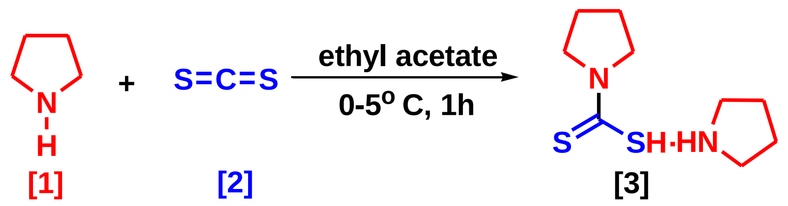

Figure S1: Synthesis of compound **pyrrolidinium pyrrolidine-1-carbodithioate**

Table S1 Spermicidal activity of reactants and product

Chemical Entity	Spermicidal MEC
Pyrrolidine [1]	~150 mM
Carbon disulfide [2]	35 mM
Pyrrolidinium pyrrolidine-1-carbodithioate [3]	0.145 mM

It is interesting to note that PPC molecule was synthesized from almost inactive reactants. With the formation of active thiol during reaction potent spermicidal activity was introduced in the product (PPC), which was 200-1000 fold more than that of the reactants.

The physicochemical data of the synthesized compound (PPC) are as follows:

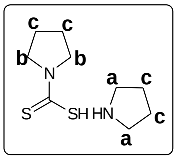

White solid, Yield 95%; mp: 182-185 °C; IR (KBr) ν (cm^{-1}): 3456 (NH), 3419 (NH), 2926 (CH_2), 1658 (C=S); ^1H NMR (300 MHz, D_2O): δ 3.65–3.58 (m, 4H, **a**), 3.19–3.11 (m, 4H, **b**), 1.88–1.81 (m, 8H, **c**); Analysis calculated for $C_9H_{18}N_2S_2$: C, 49.50; H, 8.31; N, 12.83;; found, C, 49.58; H, 8.15; N, 12.63.

Synthesis of PPC derivatives (BPC, IPC, HPC)

The PPC derivatives were synthesized according to Figure S3 as detailed below:

Benzyl pyrrolidine-1-carbodithioate (BPC) To a mixture of pyrrolidinium pyrrolidine-1-carbodithioate (3.83 mmol) and triethylamine (5.17 mmol) in methanol (25 mL) benzyl chloride (3.4 mmol) was added and stirred at room temperature for 3 h. Reaction mixture was concentrated under reduced pressure, extracted with EtOAc (2 X 20.0 mL), washed with water (2 X 5.0 mL) and the organic layer was separated. Organic layer was dried over sodium sulphate and concentrated under reduced pressure to give the pure BPC.

Physicochemical characteristics: The compound was obtained as light yellow oil, Yield 55%; IR(neat) ν (cm^{-1}); 2923, 2858, 1638, 1545, 1457, 1428, 1217; ^1H NMR (300 MHz, $CDCl_3$) δ 7.39-7.23 (5H, m), 4.58 (2H, s), 3.93 (2H, t, J = 6.7 Hz), 3.61 (2H, t, J = 6.6 Hz), 2.05-1.92 (4H, m); ESI-MS m/z 238 (MH^+). Analysis calculated for $C_{12}H_{15}NS_2$: C, 60.72; H, 6.37; N, 5.90; found: C, 60.88; H, 6.51; N, 5.99.

Compounds **IPC and HPC** were synthesized by following similar procedures.

Isobutyl pyrrolidine-1-carbodithioate (IPC) light yellow oil, Yield 65%; IR(neat) ν (cm^{-1}); 2957, 2868, 1657, 1248; ^1H NMR (300 MHz, $CDCl_3$) δ 3.99-3.93 (2H, m), 3.68 (2H, t, J = 6.7 Hz), 3.23 (2H, d, J = 6.8 Hz), 2.11-1.96 (5H, m),1.04 (6H, d, J = 6.7 Hz); ESI-MS m/z 204(MH^+). Analysis calculated for $C_9H_{17}NS_2$: C, 53.15; H, 8.43; N, 6.89; found: C, 53.29; H, 8.56; N, 6.97.

2-hydroxyethyl pyrrolidine-1-carbodithioate (HPC) light yellow oil, Yield 69%; IR(neat) ν (cm^{-1}); 3368, 2930, 1643, 1276; ^1H NMR (300 MHz, $CDCl_3$) δ 3.96-3.88 (4H, m), 3.70 (2H, t, J = 6.8 Hz), 3.59 (2H, t, J = 5.91Hz), 2.62 (1H, bs), 2.14-1.97(4H, m); ESI-MS m/z 192 (MH^+). Analysis calculated for $C_7H_{13}NOS_2$: C, 43.95; H, 6.85; N, 7.32; found: C, 43.75; H, 6.69; N, 7.18.

Figures showing reaction of PPC and its derivatives with sperm proteins

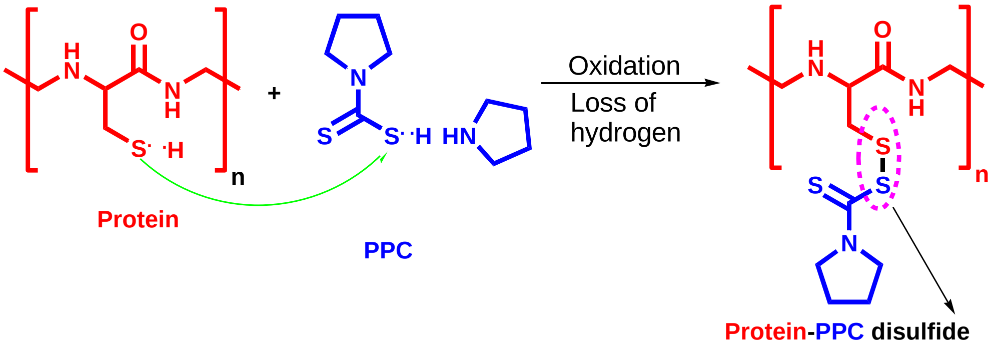

Figure S2: Proposed reaction of PPC with protein thiols

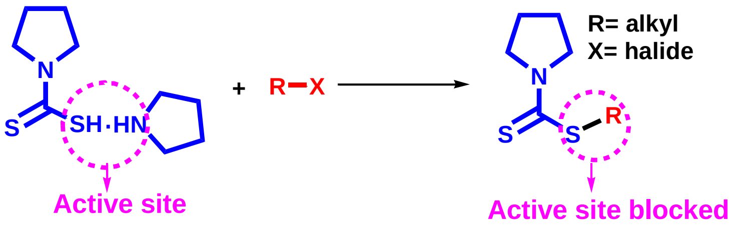

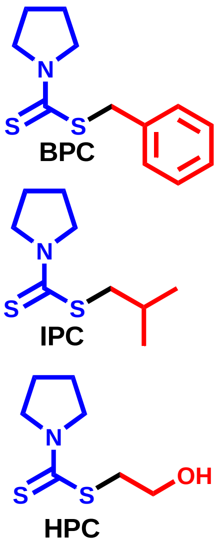

Figure S3: Preparation of PPC derivatives

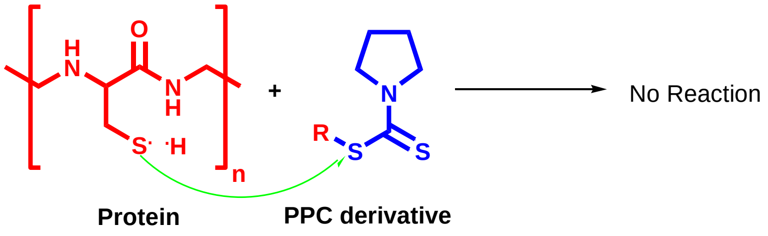

Figure S4: Reaction of PPC derivatives with protein

^{1}H, NMR of compounds BPC, IPC and HPC

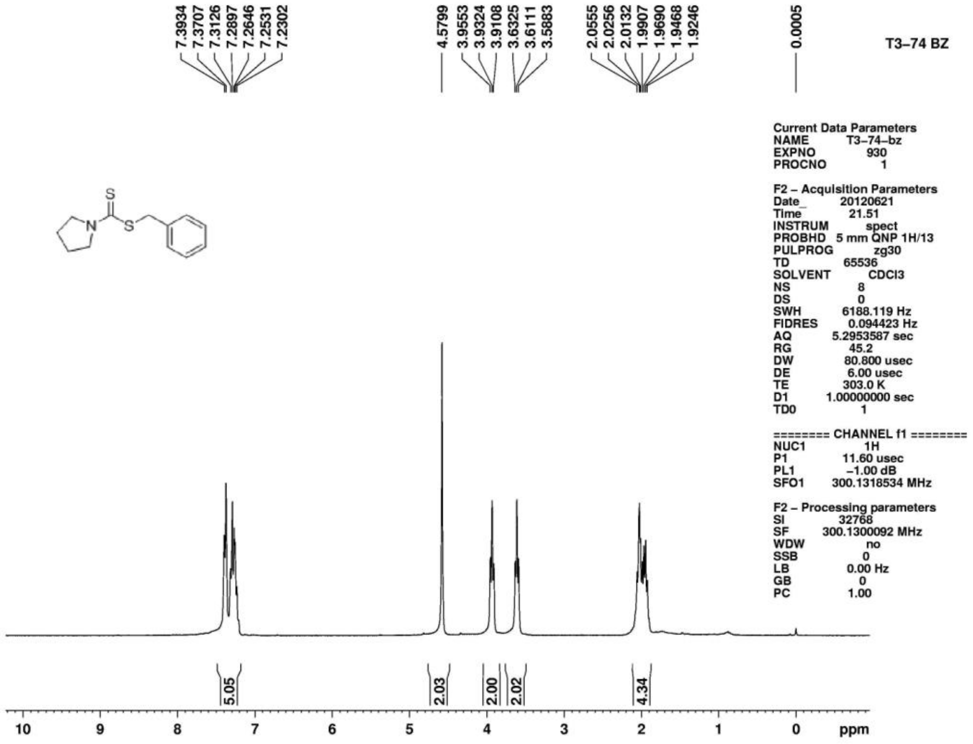

Figure S5: ^{1}H NMR of BPC at 300 MHz ($CDCl_3$)

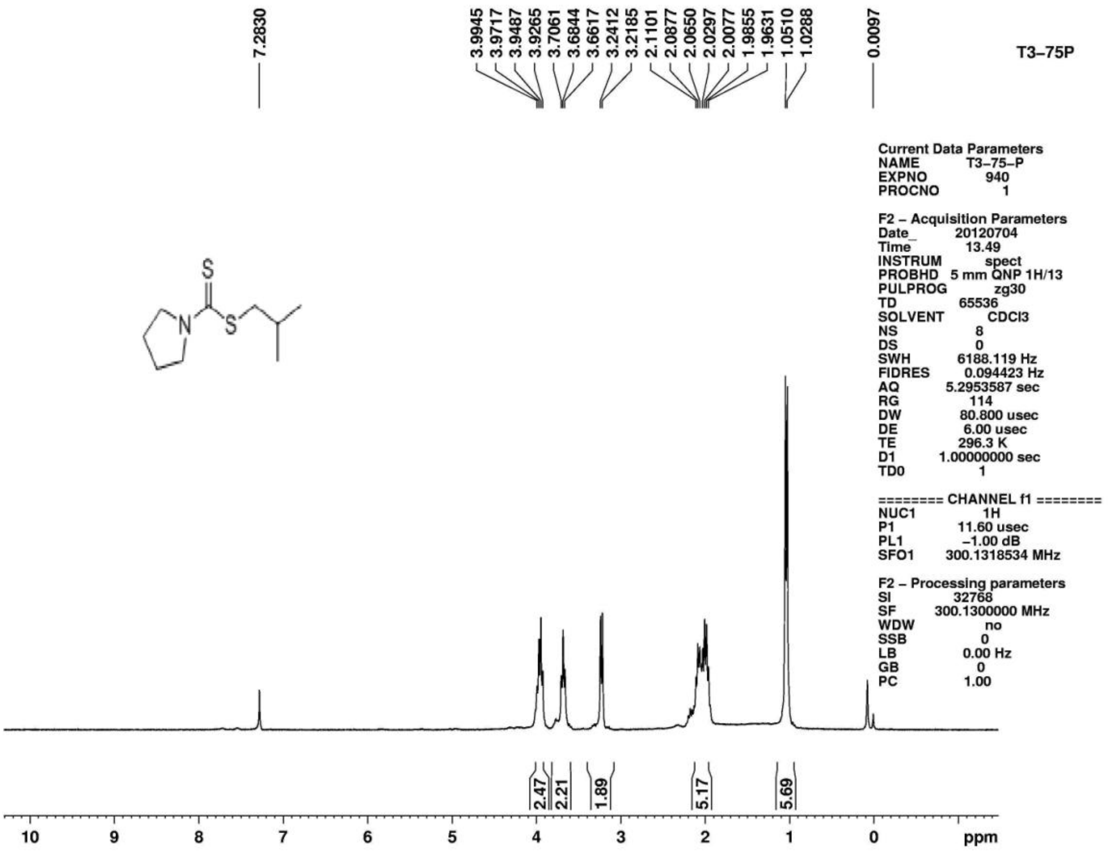

Figure S6: ^{1}H NMR of IPC at 300 MHz ($CDCl_3$)

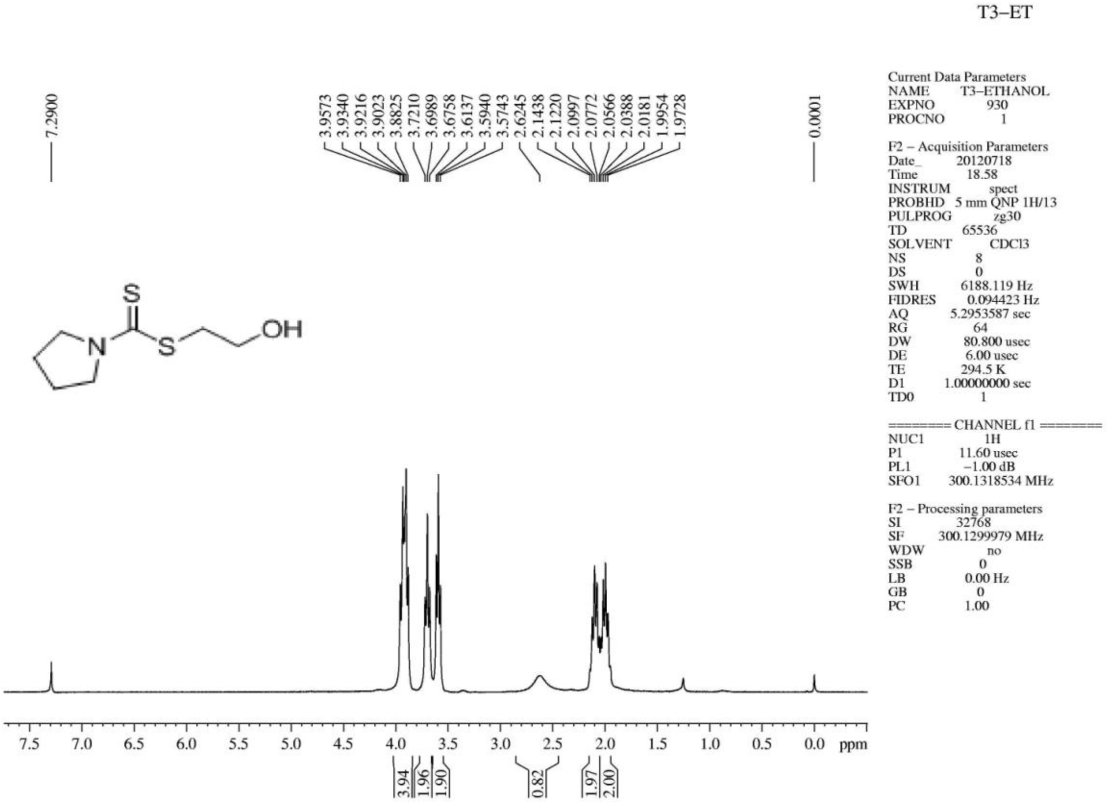

Figure S7: ^{1}H NMR of HPC at 300 MHz ($CDCl_3$)

Table S2 **Optimization of film formulation for PPC**

**Formulation codes**	**Polymer combination**	**Plasticizer**	**Composition (CH:HEC:HPMC:PVA)**	**Plasticizer concentration in solution (% w/w)**	**Physical characteristics of the film**
**A1**	CH	PEG 400	1:0:0:0	5.64	Stiff and opaque, difficult to remove from plate
**A2**	CH-HEC	PEG 400	3:1:0:0	5.64	Stiff and opaque, peelable
**A3**	CH-HEC	PEG 400	1:3:0:0	5.64	Less Stiff and opaque, peelable
**A4**	CH-HEC	PEG 400	1:1:0:0	5.64	Low flexibility, opaque, peelable
**A5**	CH+PVA	PEG 400	3:0:0:1	5.64	Papery and translucent, peelable
**A6**	CH+PVA	PEG 400	1:0:0:1	5.64	Papery and translucent, peelable
**A7**	CH-HEC-PVA	PEG 400	1:2:0:1	5.64	Papery and translucent, easily peelable
**A8**	HEC	PEG 400	0:1:0:0	5.64	Very soft, sticky, transparent, difficult to remove from plate.
**A9**	HEC-PVA	PEG 400	0:1:0:1	2.25	Soft, low flexibility transparent, peelable
**A10**	HEC-PVA	Glycerol	0:1:0:1	2.25	Colourless with drug precipitates visible on surface, difficult to remove from plate
**A11**	HEC-PVA-HPMC	Glycerol	0:1:1: 0.01	5	Colourless and clear, difficult to remove from plate
**F1**	HEC-PVA-HPMC	PEG 400	0:1:1: 0.01	5	Soft, flexible and transparent, easily peelable
**F2**	HEC-HPMC	PEG 400	0:1:1:0	2.25	Soft, flexible and transparent, easily peelable

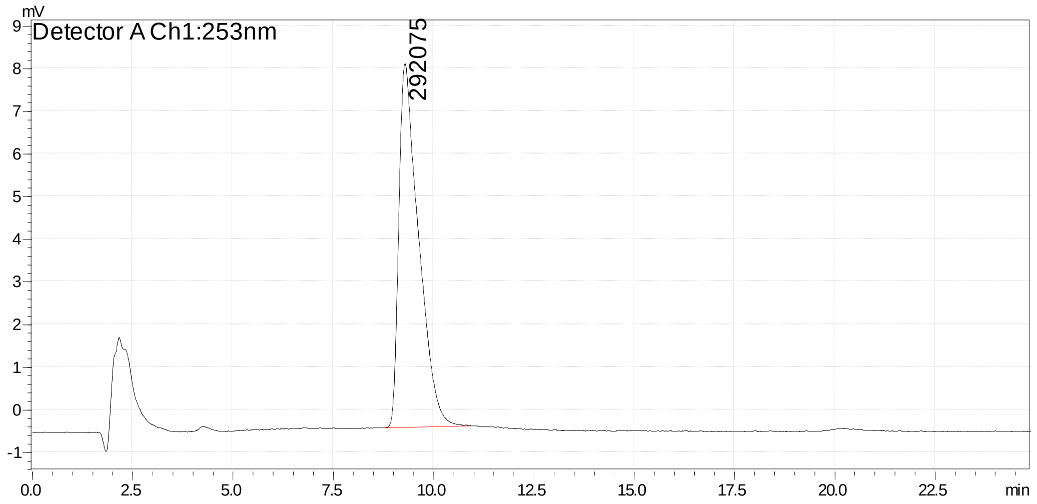

Figure S8: A typical HPLC chromatogram of PPC
